# O.4.6-4 Engagement and knowledge in a participatory systems approach to promote physical activity and mental health at vocational schools in Denmark: the Data Health study

**DOI:** 10.1093/eurpub/ckad133.223

**Published:** 2023-09-11

**Authors:** Charlotte Demant Klinker, Adrian Bauman, Anne-Louise Bjerregaard, Rikke Fredenslund Krølner, Clara Heinze

**Affiliations:** Steno Diabetes Center Copenhagen, Denmark; Sydney University, Australia; Steno Diabetes Center Sjælland, Denmark; National Institute of Public Health, Denmark; charlotte.demant.klinker@regionh.dk; Steno Diabetes Center Copenhagen, Denmark; National Institute of Public Health, Denmark; charlotte.demant.klinker@regionh.dk

## Abstract

**Purpose:**

Strong community engagement and capacity have been associated with positive intervention outcomes in community-based participatory health promotion interventions. The Data-driven Health Promotion at Vocational schools (Data Health) study aims to catalyze systems change in vocational school settings through community-led collective health promoting actions targeting physical activity and mental health among students. Three Group Model Building (GMB 1-3) sessions were delivered at eight intervention sites to build capacity and engage schools, municipalities, and communities. As part of the process evaluation, we assessed level of stakeholder knowledge, used as an indicator for capacity, and engagement prior to implementation of actions.

**Methods:**

Data were collected among participants at GMB-3 during 2022 using a modified version of the validated Stakeholder-driven Community Diffusion Survey assessing two factors: knowledge (eight items) and engagement (16 items). Five-point Likert scale scores were recoded to values between 0 (strongly disagree) to 1 (strongly agree) and means for each factor were calculated. T-tests and one-way ANOVA were used to compare level of knowledge and engagement by type of community actor and health theme.

**Results:**

Across intervention sites, 120 completed the survey; >95% of all participants. Four sites (71 respondents) had chosen mental health as their focus area prior to GMB-3 while four sites had chosen physical activity (49 respondents). Most respondents were students (58%), followed by Civic society actors (19%), vocational school staff (19%), Municipal staff (6%) and vocational management (3%) (2% missing). Mean level of engagement was 0.89 and mean level of knowledge was 0.78. Significant differences were found for both factors by chosen health theme (with lower levels at mental health sites, p < 0.05) and type of community actor (with lower levels among student and municipal staff, p < 0.001).

**Conclusions:**

High levels of knowledge among local stakeholders are needed to understand and support diffusion of evidence-informed interventions through the community; high levels of engagement are a prerequisite for inducing collaborative and sustainable efforts. Assessment of knowledge and engagement prior to implementation of actions is important to understand outcomes across intervention sites in the Data Health study.

**Support/Funding Source:**

The Danish Regions, Tværspuljen, Helsefonden, University of Southern Denmark, in-kind contributions.

